# Docosahexaenoic Acid Supplementation Does Not Improve Western Diet-Induced Cardiomyopathy in Rats

**DOI:** 10.1371/journal.pone.0051994

**Published:** 2012-12-26

**Authors:** Kimberly M. Jeckel, D. N. Rao Veeramachaneni, Adam J. Chicco, Phillip L. Chapman, Christopher M. Mulligan, Jennifer R. Hegarty, Michael J. Pagliassotti, Lindsay A. Ferguson, Gerrit J. Bouma, Melinda A. Frye

**Affiliations:** 1 Department of Biomedical Sciences, College of Veterinary Medicine and Biomedical Sciences, Colorado State University, Fort Collins, Colorado, United States of America; 2 Department of Health and Exercise Science, College of Applied Human Sciences, Colorado State University, Fort Collins, Colorado, United States of America; 3 Department of Statistics, College of Natural Sciences, Colorado State University, Fort Collins, Colorado, United States of America; 4 Department of Food Science and Human Nutrition, College of Applied Human Sciences, Colorado State University, Fort Collins, Colorado, United States of America; 5 Department of Chemical and Biological Engineering, College of Engineering, Colorado State University, Fort Collins, Colorado, United States of America; 6 Creighton University School of Medicine, Omaha, Nebraska, United States of America; 7 Department of Clinical Sciences, College of Veterinary Medicine and Biomedical Sciences, Colorado State University, Fort Collins, Colorado, United States of America; University of Otago, New Zealand

## Abstract

Obesity increases risk for cardiomyopathy in the absence of hypertension, diabetes or ischemia. The fatty acid milieu, modulated by diet, may modify myocardial structure and function, lending partial explanation for the array of cardiomyopathic phenotypy. We sought to identify gross, cellular and ultrastructural myocardial changes associated with Western diet intake, and subsequent modification with docosahexaenoic acid (DHA) supplementation. Wistar and Sprague-Dawley (SD) rats received 1 of 3 diets: control (CON); Western (WES); Western + DHA (WES+DHA). After 12 weeks of treatment, echocardiography was performed and myocardial adiponectin, fatty acids, collagen, area occupied by lipid and myocytes, and ultrastructure were determined. Strain effects included higher serum adiponectin in Wistar rats, and differences in myocardial fatty acid composition. Diet effects were evident in that both WES and WES+DHA feeding were associated with similarly increased left ventricular (LV) diastolic cranial wall thickness (LVW_cr/d_) and decreased diastolic internal diameter (LVID_d_), compared to CON. Unexpectedly, WES+DHA feeding was associated additionally with increased thickness of the LV cranial wall during systole (LVW_cr/s_) and the caudal wall during diastole (LVW_ca/d_) compared to CON; this was observed concomitantly with increased serum and myocardial adiponectin. Diastolic dysfunction was present in WES+DHA rats compared to both WES and CON. Myocyte cross sectional area (CSA) was greater in WES compared to CON rats. In both fat-fed groups, transmission electron microscopy (TEM) revealed myofibril degeneration, disorganized mitochondrial cristae, lipid inclusions and vacuolation. In the absence of hypertension and whole body insulin resistance, WES+DHA intake was associated with more global LV thickening and with diastolic dysfunction, compared to WES feeding alone. Myocyte hypertrophy, possibly related to subcellular injury, is an early change that may contribute to gross hypertrophy. Strain differences in adipokines and myocardial fatty acid accretion may underlie heterogeneous data from rodent studies.

## Introduction

Obese individuals are at higher risk for the development of cardiomyopathy leading to heart failure.[1] Within the obese population, these structural and functional changes of the heart may represent a greater risk for death than does coronary artery disease.[2] Obesity-mediated cardiomyopathy (OC) and heart failure have traditionally been attributed to hypertension, myocardial ischemia and diabetes. More recently, increased LV mass and myocardial dysfunction have been associated with obesity in otherwise healthy humans, in the absence of these comorbidities.[3]–[7] Often present in healthy obese individuals,[4], [5], [7], [8] LV hypertrophy (LVH) is an early echocardiographic change that reflects increased LV mass. Importantly, LVH is an independent risk factor for development of systolic dysfunction,[9] and is associated with an increased risk for cardiovascular and all-cause mortality.[10]–[12] Collectively, this information suggests that early obesity may not be entirely benign, and that important cardiomyopathic changes occur in the absence of conventional comorbidities.

There is evidence that the fatty acid milieu predicts structural and functional changes in the heart that occur with obesity. Dietary supplementation with omega-3 (n-3) polyunsaturated fatty acids (PUFA) attenuates pathologic remodeling in response to pressure overload,[13] myocardial ischemia[14] and treatment with rosiglitazone.[15] Studies of LVH in response to hypercaloric feeding alone have been conducted;[16], [17] however, much remains to be learned about the cellular changes contributing to LVH in uncomplicated obesity, and how these changes might be modulated by dietary n-3 supplementation.

The data generated from rodent dietary studies are diverse and often inconsistent. Published work in both rats and mice suggest that dietary obesity involves an interaction between strain and diet,[18], [19] and that Wistar and SD strains specifically display distinct lipid metabolism.[20] We therefore studied both Wistar and SD rats, 2 strains commonly used in dietary obesity studies, to define strain differences that may contribute to unique dietary responses.

Consumption of a Western diet places an individual at greater risk for development of obesity,[21], [22] and is associated with cardiomyopathic changes in obese rodents with concomitant insulin resistance and hypertension.[23] We sought to investigate the contribution of Western diet intake to early cardiac outcomes preceding overt obesity, and of dietary fatty acid composition and strain to the heterogeneous array of cardiac phenotypy. Accordingly, we determined whether LVH occurs in association with intake of a Western diet, in the absence of hypertension or apparent insulin resistance, and whether concomitant supplementation with dietary DHA modified any hypertrophic response. We also sought to identify strain differences that may contribute to divergent results of rodent dietary studies. Primary outcomes included myocardial structure and function, and underlying processes possibly contributing to LV thickening: ECM remodeling, myocardial lipid accumulation and cardiac myocyte hypertrophy. Secondary outcomes included adiposity, hemodynamics and serum metabolic indices, as well as myocardial protein carbonylation, adiponectin content and fatty acid composition.

## Methods

### Ethics Statement

Protocols and conditions meet or exceed standards as described in the Animal Welfare Act regulations, the Guide for the Care and Use of Laboratory Animals and the Guide for the Care and Use of Agricultural Animals in Agricultural Research and Teaching. The Colorado State University Institutional Animal Care and Use Committee specifically approved this study.

### Animals

Adult male Wistar and SD rats (Charles River Laboratories, Wilmington, MA) were maintained in the Colorado State University Laboratory Animal Resource Center in a temperature- and humidity-controlled environment. Rats were housed in pairs with a normal 12-hour light/12 hour dark cycle. The rats were allowed a 2-week acclimation period prior to initiation of dietary treatment.

### Diet

A representative Western diet is characterized by increased saturated fat, a high (10∶1–30∶1) ratio of n-6∶n-3 PUFA and increased simple carbohydrate content. Compared to the control diet, our Western diet was formulated to reflect these differences, with a saturated fat content of 69% total fatty acids (compared to 48% in the control diet) and an n-6∶n-3 of 21∶1. Additionally, the Western diet contained the simple carbohydrate sucrose along with the complex carbohydrate cellulose, whereas the control diet contained only complex carbohydrates (cellulose and cornstarch). Diets were supplied by Harlan Teklad (Madison, WI) and are detailed in Tables 1 (macronutrient composition, caloric density) and S1 (fatty acid composition). Dietary DHA was in the form of microalgae-derived DHASCO® oil (Martek, Columbia, MD), and was mixed into the diet during manufacturing. A previous study of DHASCO® oil consumption in rats revealed no adverse outcomes at doses exceeding those used in the present study.[24] Further, dietary DHA content was lower than that used in some studies of DHA and cardiovascular outcomes[25] and similar to that used in others.[26] Studies investigating effects of fish oil, which contains both DHA and eicosapentaenoic acid (EPA), commonly incorporate less DHA than was used in the present study.

**Table 1:** Macronutrient composition and caloric density of diets.

	CON	WES	WES+DHA
Protein % kcal	16.4	16.4	16.4
Carbohydrate % kcal	72.8	25.8	25.8
Fat % kcal	10.8	57.9	57.9
Saturated fat % total fatty acids	48.0	69.0	69.0
Monounsaturated fat % total fatty acids	21.0	21.0	21.0
Polyunsaturated fat % total fatty acids	31.0	10.0	10.0
Kcal/g	3.6	4.5	4.5

% kcal, % of total diet kcal; CON, control; WES, Western; WES+DHA, Western + DHA.

At 6 weeks of age, Wistar and SD rats were divided into 1 of 3 dietary treatment groups (6 groups total; n = 10/strain/treatment): control (CON), Western (WES) and Western + DHA (WES+DHA). Previous work showed that male Wistar rats fed diets enriched in α-linolenic acid (ALA) and DHA had stable myocardial fatty acid composition after 2 months of dietary treatment.[26] With 16 weeks of high-fat, high-carbohydrate feeding, Wistar rats developed myocardial hypertrophy and dysfunction, but with accompanying hemodynamic and metabolic aberrancy.[23], [27] In an effort to efficiently produce LVH while limiting development of comorbidities, the duration of dietary treatment in the present study was 12 weeks. Food intake and body weights were measured twice weekly. Rats were fasted overnight prior to terminal sample collection. Feed efficiency was calculated using the following formula: [gm weight gain∶kcal consumed]×100.

### General anesthesia

General anesthesia was induced by placing rats in a commercial rodent anesthesia chamber and initiating flow of 3% isofluorane in a 95% O_2_/5% CO_2_ gas mixture. Anesthesia was maintained by nosecone at 2% isofluorane for noninvasive measurements, and at 4% isofluorane for terminal sample collection. A surgical plane of anesthesia was confirmed when a bilateral toe pinch failed to elicit limb withdrawal or change in respiratory rate/pattern.

### Systolic blood pressure and heart rate

Immediately upon moving to the maintenance dose of 2% isofluorane, measurement of heart rate (HR) and systolic blood pressure (SBP) was accomplished using the tail cuff method (SC 1000 Pressure Analysis System, Hatteras Instruments, Cary, NC). To reduce inherent variability and optimize pulse detection,[28], [29] rats were maintained on a temperature-controlled platform prior to and during measurement. Three separate measurements of both HR and SBP were recorded, and averaged for each rat. If 3 consecutive readings were not obtained, the data were omitted from the statistical analysis.

### Echocardiographic examination

After HR and SBP determination, rats were shaved over the ventral thorax and upper abdomen. A Philips HD-11 ultrasound machine with a 12 mHz pediatric sector transducer was used to image the heart in transverse parasternal and 4-chamber views. Two dimensional and M-mode imaging was incorporated to measure LV end-diastolic and end-systolic wall and chamber dimensions, and the % fractional shortening (FS) was derived as follows: %FS = [LVID_d_−LVID_s_/LVID_d_]×100, where LVID_s_ = LV internal diameter during systole. Doppler imaging was used to measure isovolumic relaxation time (IVRT), an index of diastolic function (ability of the ventricles to relax and fill). Left ventricular mass was estimated using a formula adapted from Foppa et al: [30] LV mass = 0.8 (1.04 ([LVID_d_+LVW_cr/d_+LVW_ca/d_]^3^−[LVID_d_]^3^))+0.6 g.

### Processing of tissue samples

The heart was exposed through a medial sternotomy. Exsanguination was accomplished by aspirating approximately 6 mL of blood from the pulmonary arterial trunk. Blood was immediately placed into a collection tube and after 2 hours, was centrifuged at 2095 RCF for 15 minutes. After centrifugation, serum was aspirated and stored at −80°C.

Immediately upon withdraw of the blood sample, the heart was excised and placed in ice cold saline, then quickly dabbed for excess fluid prior to recording of heart weight. While in the iced saline, the heart was dissected to isolate the LV, right ventricle and septum, and isolated tissue weights were recorded. Samples of right and left ventricular and septal myocardium were divided and either snap frozen in liquid nitrogen and stored at −80°C or fixed in 4% paraformaldehyde. After 24 hours in paraformaldehyde, tissues were transferred to 70% ethanol, then trimmed and embedded in paraffin. The mass of visceral adipose tissue was estimated by removing and weighing mesenteric fat.

### Serum measurements

Serum testing was completed at the University of Colorado Hospital Clinical and Translational Research Center. Serum leptin and adiponectin were measured using the Rat Leptin RIA and Human Adiponectin RIA Kits, respectively (Millipore, St. Charles, MO). Insulin was quantitated using a commercial rat insulin ELISA (Linco Research, St. Charles, MO). Glucose and free fatty acids (FFA) were measured using the Roche Cobas Mira Plus Chemistry Analyzer (Indianapolis, IN). The Homeostasis Model Assessment (HOMA) was used to estimate insulin resistance using a HOMA2 IR Excel-based calculator (http://www.dtu.ox.ac.uk/homacalculator/download.php). Serum triglycerides (TG) were assayed using enzymatic colorimetry (Triglycerides Reagent, Thermo Electron, Pittsburg, PA).

### Determination of protein oxidation

To conserve resources, only myocardial tissues from Wistar rats were used for Oxyblot experiments. To test whether an increase in highly unsaturated fatty acids in the myocardial phospholipid fraction may increase susceptibility to oxidative stress, protein carbonylation was quantitated using the Oxyblot Protein Oxidation Detection Kit (Millipore, Billerica, MA). Coomassie stained gels were used to establish uniformity of protein loading. Repeated experiments using random sampling resulted in thirteen distinct data sets (CON, WES, WES+DHA) of sufficient quality for analysis, derived from 7 membranes.

### Real time reverse transcription polymerase chain reaction (RT-PCR)

Tissue from Wistar rats was included in RT-PCR experiments aimed at measuring myocardial adiponectin gene expression. RNA isolation and reverse transcription: Total RNA was isolated from rat myocardial tissue using TRIzol Reagent (Life technologies, Grand Island, NY), followed by treatment with RNase-free DNase (QIAGEN, Valencia, CA). Sample RNA concentration was determined using a NanoDrop ND-100 Spectrophotometer and RNA integrity was analyzed on the Agilent 2100 Bioanalyzer. Messenger RNA (one microgram) was reverse transcribed into cDNA using a Quanta Biosciences cDNA Synthesis kit (Quanta Biosciences Inc., Gaithersburg, MD) according to manufacturer's instructions.

#### Primer design and validation

Primer pairs were designed for the *Adipoq*, *Rn18s* and *Gapdh* genes using Primer3 program (http://frodo.wi.mit.edu/), and the sequences are provided in Table S2. Primer specificity was confirmed based on PCR amplification of a single band followed by sequencing of the amplicons. PCR amplification efficiencies were determined using cDNA produced from pooled RNA samples of myocardial tissue, and generation of standard curves based on ten-fold serial dilutions of the cDNA.

#### Real-time RT-PCR

To determine relative levels of *Adipoq*, real-time PCR analysis was performed using SYBR Green (Roche Applied Science, Indianapolis, IN). Two housekeeping genes, *Rn18s* and *Gapdh*, were included as internal controls. A melt curve analysis was performed to confirm the amplification of a single PCR product.

Relative levels of *Adipoq* were determined by normalizing the raw Cp values using the geometric mean of *Rn18s* and *Gapdh* as a normalization factor. Relative levels are presented as the mean Cp values relative to the normalization factor for each treatment group.

### Immunoblot analysis

Tissue from Wistar rats was used for Western blotting experiments aimed at quantitating myocardial adiponectin protein. Frozen LV tissue was pulverized under liquid nitrogen, then homogenized in a lysis buffer containing 20 mM HEPES (pH 7.4), 1% Triton X-100, 10% glycerol, 2 mM EGTA, 1 mM sodium vanadate, 2 mM dithiothreitol, 1 mM phenylmethylsulfonyl fluoride, 50 mM β-glycerophosphate, 3 mM benzamidine, 10 µM leupeptin, 5 µM pepstatin and 10 µg/mL aprotinin. Equal volumes of homogenate containing 50 µg of protein were separated by molecular weight using SDS-PAGE, then transferred to a polyvinylidene fluoride membrane (Hybond-P, GE Healthcare, Pittsburgh, PA). The membrane was incubated in a 1∶200 solution of antibody directed against adiponectin (Abcam, Cambridge, MA), followed by a 1∶5000 solution of horseradish peroxidase-conjugated secondary antibody (Santa Cruz Biotechnology, Santa Cruz, CA). After application of a chemiluminescent reagent (ImmunoCruz, Santa Cruz Biotechnology, Santa Cruz, CA), signal intensity was quantitated using a UVP imaging system (UVP, Upland, CA). Two experiments were conducted, each incorporating tissue from 3 randomly selected rats from each treatment group (n = 6).

### Myocardial collagen and triglyceride; myocyte area

Left ventricular thickening attributable to high fat feeding or obesity is most often attributed to extracellular matrix (ECM) remodeling, myocardial lipid accumulation and/or cardiac myocyte hypertrophy. There is evidence that these processes are differentially affected by dietary fat, so were chosen for emphasis in the present study.

Masson's trichrome stain was used for collagen detection in paraffin-embedded tissue sections. A slide from each animal was evaluated at 20× for regions of transversely sectioned LV cells without artifact or large vessels. Four images per slide, comprising identical total areas among slides, were assessed for % total area that was positive for Masson's staining, using NIH Image J software (Rasband, W.S., ImageJ, U.S. National Institutes of Health, Bethesda, Maryland, USA, http://imagej.nih.gov/ij/, 1997–2012). Hydroxyproline (a primary amino acid in collagen) was quantitated in frozen septal tissue spectrophotometrically using previously described methods.[31]

Based on data from previous studies, a sample size of 4 was used to characterize the myocardial fatty acid profile. Lipids were extracted from frozen septal tissue from randomly selected animals using the method described by Matyash et al.[32] Briefly, tissue samples were pulverized into a powder under liquid nitrogen and placed in a glass homogenizer. Methanol (0.75 mL) and methyl-tert-butyl ether (2.5 mL) were added and the powdered tissue was homogenized on ice. Samples were capped off under nitrogen gas and incubated at room temperature for one hour, then purified water was added. After vortexing, samples were capped off under nitrogen and incubated at room temperature for 10 minutes, then centrifuged at 1,000 RCF for 10 minutes. The upper (organic) phase was collected and the sample dried under nitrogen. Samples were frozen at −80°C until processed. Thin layer chromatography was used to separate out the phospholipid fraction using a 20 cm×20 cm silica gel plate in a 70∶30∶1 hexane∶ethyl ether∶acetic acid solution. The band associated with the phospholipid fraction was scraped from the plate and dissolved in hexane and KOH. Three ml of 14% boron trifluoride in methanol was added and each sample was placed on a heat plate at 70°C for 1.5 hours to obtain methyl esters. The analysis was performed using an Agilent 6890 Series Gas Chromatographer (Agilent Technologies, Inc., Santa Clara, CA). The column used was an Agilent Technologies DB-225 30 m×0.250 mm×0.25 µm, model 122–2232. The initial temperature of the oven was 100°C with an initial ramp temperature of 10°C/min for 10 minutes, then 2.5°C/min for 4 minutes and held at 210°C for the remaining 15 minutes for a total run time of 29 minutes. The inlet split ratio was 20∶1 with the column at constant flow and an initial flow, pressure and velocity at 2.0 ml/min, 23.86 psi and 44 cm/sec, respectively.

Septal TG were extracted from remaining tissue samples (n = 5–8) and quantitated using the commercial colorimetric Triglyceride Quantification Kit (BioVision Research Products, Mountain View, CA).

Myocyte CSA was measured in hematoxylin- and eosin-stained LV sections, using NIH Image J software. Fifty transversely sectioned cells with central nuclei from each of 2 slides per rat were evaluated (i.e. 100 cells/rat).

### Transmission electron microscopy

A sample size of 2 was chosen due to limited resources. Left midventricular tissue samples (2 mm^3^) from randomly chosen Wistar rats from each treatment group were fixed in 2% glutaraldehyde with post-fixation in 1% osmium tetroxide (in 0.1 M cacodylate buffer), and embedded in Poly/Bed 812 (Polysciences, Warrington, PA, USA). A series of 1-µm-thick sections were cut, stained with toluidine blue for light microscopy, and longitudinally oriented cardiac myocytes were marked for TEM. Thin sections (60–80 nm) were cut from the identified areas of Poly/Bed-embedded tissues, stained with uranyl acetate and lead citrate and examined using a transmission electron microscope (JEOL-1200EX, JEOL USA, Inc., Peabody, Massachusetts).

### Statistics

Based on previous studies, the echocardiographic parameter of LV wall thickness was chosen as the principle outcome measurement. A power calculation was conducted using software developed by R.V. Lenth (Lenth, R. V., Java Applets for Power and Sample Size. http://www.stat.uiowa.edu/~rlenth/Power). A one-way ANOVA was used to estimate the within group variance as 0.0281 and suggest a hypothesized difference between LV wall thickness as 0.047. Using these estimates, power for a 2-sample, 2-sided t-test (α = 0.05) was calculated as 0.9421 with a sample size of 10 per group.

Statistical analyses were conducted using JMP Pro 9.0.2 (SAS Institute Inc., Cary, NC). Treatment and strain effects were compared using 2-way ANOVA. When the interaction F-test p-value was >0.05, diets were compared separately within strain, and strains were compared separately within diet. The LSD method for pairwise comparisons was used when diet or strain F-test p-values were <0.05. Data are expressed as mean +/− SE; statistical significance was set at p<0.05. The homogeneity of variance assumption of the ANOVA model was assessed by inspecting plots of residuals versus predicted values. Datasets with evidence of unequal variance were first log transformed. If unequal variance persisted, diet and strain effects were assessed on raw data using Welch's ANOVA and t-test, respectively. Normality of the residuals was evaluated using the Shapiro-Wilk test. The Kruskal-Wallis (diet) and Mann Whitney (strain) nonparametric tests were used to analyze non-normal data. Pearson's and Spearman's correlations were applied to normal and non-normal data, respectively. Outliers were identified using the Grubbs test, with calculations performed online at http://www.graphpad.com/quickcalcs/Grubbs1.cfm.

Outcomes compared within 1 strain (protein carbonylation, adiponectin RT-PCR and immunoblotting) were subjected to identical testing of variance and distribution, and analyzed using 1-way ANOVA. The LSD method for pairwise comparisons was applied when the overall p-value was <0.05.

## Results

### Diet effects

Tabular data within the manuscript includes outcomes relevant to the 6 treatment groups (i.e. according to diet and strain). The p-values derived from 2-way ANOVA (i.e. diet, strain, interaction) are also provided. Significant diet effects are represented graphically. Supporting information includes tabular summaries of diet effects across strain (S5–S7).

#### Morphometry, HR and SBP

Data relevant to intake, body morphometry, organ weights, hemodynamics and serum metabolic indices are presented in Tables 2, S3 and S5. Averaged across intake and body weight and not accounting for feed loss in bedding, rats consumed approximately 0.26 gm DHA/day; the American Heart Association recommends that individuals consume 1–4 gm of EPA+DHA daily.[33] There was no diet effect on total energy intake, feed efficiency (Tables S3, S5), terminal body weight or weight gain (Tables 2, S5). Absolute and indexed (relative to body weight) visceral adipose, heart and LV masses were not different (Tables 2, S3 and S5). Heart rate and SBP were also similar across dietary groups (n = 4–9; Tables 2, S5).

**Table 2:** Data summary including body morphometry, tissue masses indexed to body weight, hemodynamics and selected serum metabolic indices, according to diet and strain.

	CON		WES		WES + DHA		p value (diet)	p value (strain)	p value (int)
	SD	WIS	SD	WIS	SD	WIS			
**Final body weight and indexed tissue weights**									
**Body wt (g)**	611±19	551±15	590±24	564±18	605±20	571±16	0.842	0.012	0.657
**Wt gain (g)**	413±15	353±14	386±22	366±16	399±18	374±14	0.814	0.015	0.448
**Visceral adipose: body wt**	0.0109±0.0011	0.0091±0.0005	0.0100±0.0010	0.0093±0.0009	0.0096±0.0008	0.0096±0.0009	0.873	0.238	0.579
**Heart: body wt**	0.0024±6.961e^−5^	0.0023±4.955e^−5^	0.0023±5.667e^−5^	0.0023±<0.0001	0.0023±5.385e^−5^	0.0023±5.667e^−5^	0.581	0.221	0.628
**LV:body wt**	0.0012±4.783e^−5^	0.0012±3.922e^−5^	0.0011±<0.0001	0.0011±4.018e^−5^	0.0012±2.735e^−5^	0.0012±<0.0001	0.582	0.714	0.997
**Heart rate and blood pressure**									
**HR (bpm)**	376±11.1	399±7.6	370±14.8	427±14.6	375±12.5	392±14.9	0.511	0.005	0.274
**SBP (mm Hg)**	149.9±10.40	137.8±5.66	116.8±5.67	177.0±13.15	141.0±5.63	137.8±4.67	0.750	0.073	0.001
**Serum measurements and HOMA**									
**Adiponectin (ug/mL)**	16.67±1.79	22.46±1.08	16.84±1.55	22.22±1.85	23.75±3.12	31.26±3.80	0.001	0.002	0.893
**FFA (uEq/L)**	723.2±42.0	695.4±53.0	577.0±31.5	610.1±39.1	534.3±34.6	610.4±21.6	0.002	0.394	0.411
**TG (mg/dl)**	123.8±19.9	90.7±12.0	84.5±14.6	68.3±5.6	52.1±5.9	56.3±5.0	<0.001	0.395	0.398

Data displayed as mean ± SE relevant to each treatment group (diet/strain). The p-values derived from 2-way ANOVA (representing diet, strain and interaction effects) are provided. CON, control; WES, Western; WES+DHA, Western + DHA. HR/SBP n = 4–9.

#### Serum measurements

**Figure 1:**
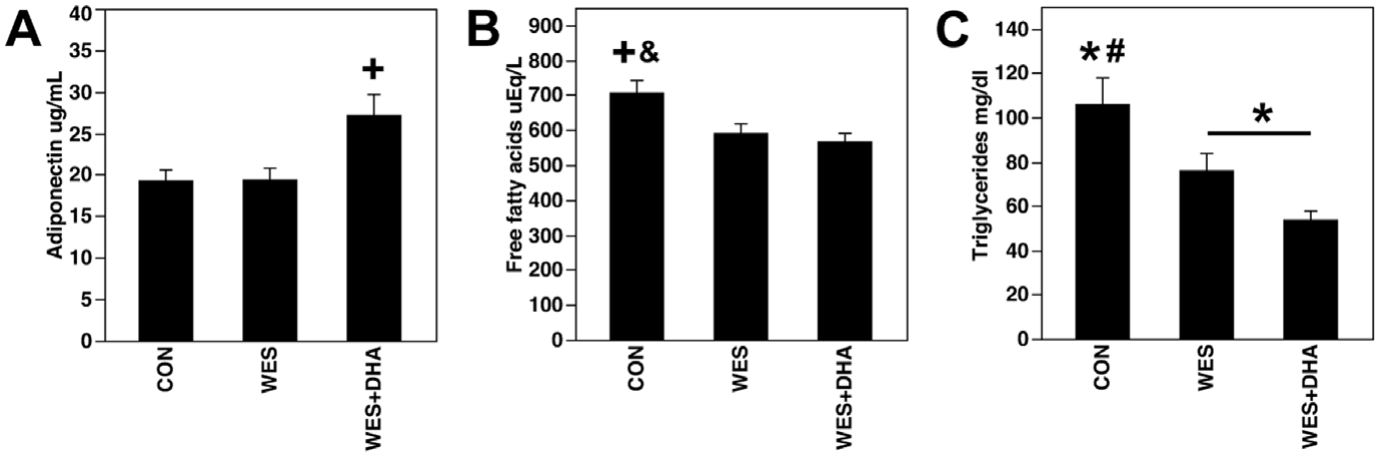
Serum adiponectin, FFA and TG concentrations. A: Rats fed the WES+DHA diet had greater serum adiponectin than CON and WES fed rats (+p<0.01 compared to both CON and WES). B,C: Serum FFA and TG were lower in both fat-fed groups compared to CON rats (*p<0.05; +p<0.01; &p<0.001 compared to CON). Further, rats fed the WES+DHA diet had lower TG concentrations than WES fed rats (C) (*p<0.05).

Serum leptin was correlated with body weight (r = 0.288, p = 0.028), absolute visceral adipose weight (r = 0.513, p<0.0001) and visceral adipose weight indexed to body weight (r = 0.521, r<0.0001). There were no differences in serum leptin according to diet (Tables S3, S5). Serum adiponectin was not correlated with body weight or absolute/indexed visceral adipose mass. Serum adiponectin was, however, increased in WES+DHA fed rats compared to both CON and WES fed rats (Figure 1A; Table S5). Fasting serum insulin and glucose and calculated HOMA were unaffected by diet (Tables S3, S5). Serum FFA and TG were lower in WES and WES+DHA rats compared to CON; further, WES+DHA rats had lower serum TG compared to WES (Figure 1B,C; Table S5).

#### Myocardial phospholipid fatty acid profiles

**Figure 2:**
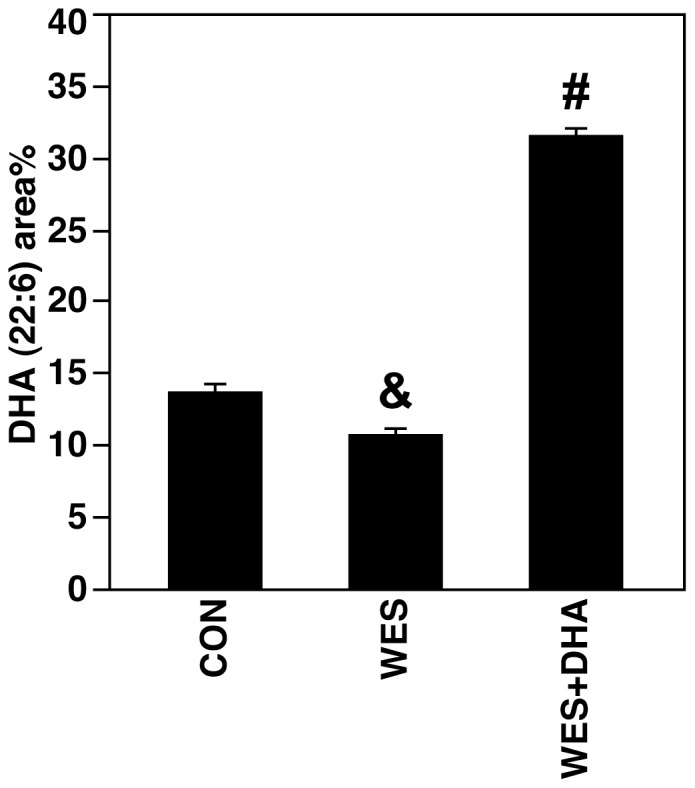
Myocardial phospholipid DHA (22:6 n-3). Rats fed the WES+DHA diet had more myocardial DHA than rats fed the CON or WES diet (# p<0.0001). Additionally, rats fed the WES diet had more myocardial DHA than CON rats (&p<0.001).

Myocardial fatty acid profiles are presented in Tables S4 and S6. There was a significant effect of diet present across all fatty acids measured. With the exception of palmitic (16∶0) and oleic (18∶1) acids, the tissue composition generally reflected direct dietary intake or intake of precursors. Importantly, myocardial phospholipid DHA (22∶6) content was greatest in WES+DHA fed rats (31.64±0.50 area %) compared to CON (13.79±0.49 area %; p<0.0001) and WES (10.82±0.43 area %; p<0.0001) (Figure 2). Rats fed the WES diet had less myocardial DHA than rats fed the CON diet (p<0.001).

#### Echocardiographic measurements

**Figure 3:**
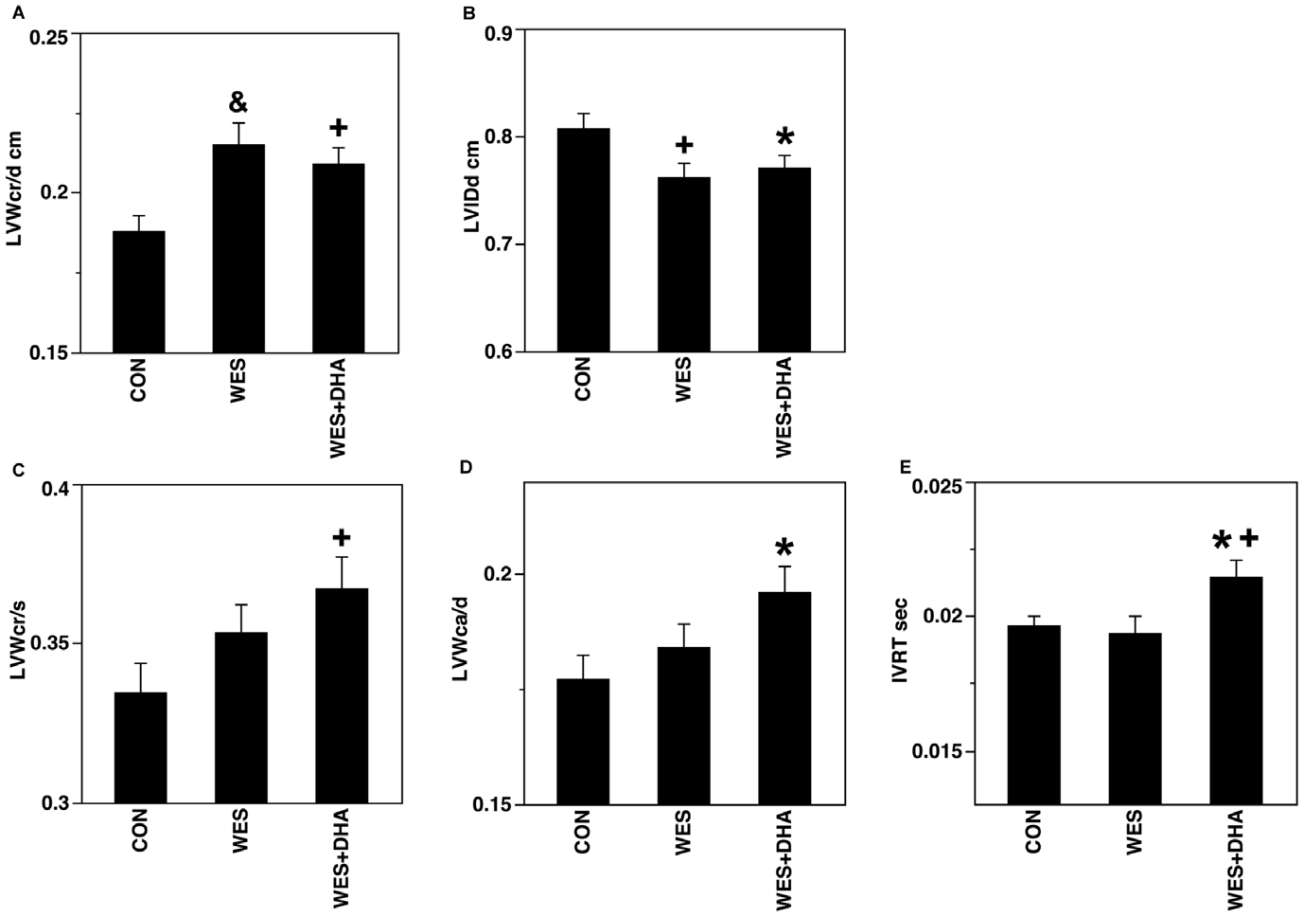
Echocardiographic indices that were different according to high-fat feeding (i.e. WES and WES+DHA; A and B) and to WES+DHA feeding alone (C–E). A,B: Both WES and WES+DHA rats had greater LVW_cr/d_ (A) and reduced LVID_d_ (B) compared to CON. C, D: WES+DHA feeding was associated with greater LVW_cr/s_ (C) and LVW_ca/d_ (D) compared to CON. E: WES+DHA rats had prolonged IVRT compared to both CON and WES. (*p<0.05; +p<0.01; &p<0.001).

Echocardiographic data are presented in Figure 3 and Tables 3 and S7. Regarding LV wall thickness, both WES (0.215±0.006 cm; p<0.001) and WES+DHA (0.209±0.005 cm; p = 0.009) rats had greater LVW_cr/d_ compared to CON (0.188±0.005 cm) (Figure 3A). Rats from both fat-fed groups also had reduced LVID_d_ (WES 0.763±0.012 cm, p = 0.009; WES+DHA 0.771±0.012 cm, p = 0.031) compared to CON rats (0.808±0.013 cm) (Figure 3B). Furthermore, WES+DHA feeding alone was associated with increased LVW_cr/s_ (0.367±0.010 cm) compared to CON rats (0.335±0.009 cm; p = 0.008) (Figure 3C), and with greater LVW_ca/d_ (0.196±0.006 cm) compared to CON rats (0.177±0.005 cm; p = 0.011) (Figure 3D). Regarding functional indices, WES+DHA fed rats had prolonged IVRT (0.0215±0.0006 sec) compared to CON (0.0197±0.0004 sec; p = 0.023) and WES (0.0194±0.0006 sec; p = 0.009) rats (Figure 3E). There were no diet effects on FS.

**Table 3:** Summary of echocardiographic measurements, myocardial hydroxyproline, collagen and triglyceride content and myocyte cross sectional area, according to diet and strain.

	CON		WES		WES + DHA		p value (diet)	p value (strain)	p value (int)
	SD	WIS	SD	WIS	SD	WIS			
**Echocardiographic measurements**									
**LVW_cr/s_ (cm)**	0.358±0.012	0.312±0.008	0.371±0.012	0.337±0.010	0.366±0.017	0.369±0.010	0.029	0.010	0.097
**LVW_cr/d_ (cm)**	0.194±0.006	0.183±0.008	0.224±0.011	0.207±0.006	0.210±0.008	0.209±0.006	0.002	0.128	0.564
**LVW_ca/s_ (cm)**	0.314±0.007	0.296±0.012	0.327±0.012	0.297±0.009	0.341±0.017	0.330±0.016	0.051	0.068	0.751
**LVW_ca/d_ (cm)**	0.181±0.007	0.174±0.007	0.195±0.006	0.174±0.007	0.204±0.007	0.188±0.008	0.035	0.018	0.605
**LVID_d_ (cm)**	0.832±0.017	0.785±0.018	0.756±0.019	0.769±0.015	0.794±0.017	0.748±0.012	0.020	0.055	0.128
**LVID_s_ (cm)**	0.420±0.019	0.411±0.018	0.363±0.035	0.393±0.019	0.388±0.038	0.334±0.016	0.108	0.615	0.280
**LV mass (g)**	1.58±0.05	1.44±0.04	1.59±0.05	1.49±0.04	1.66±0.06	1.55±0.05	0.144	0.003	0.888
**LV mass:body wt**	0.0026±0.0001	0.0026±4.568e^−5^	0.0027±0.0001	0.0026±4.795e^−5^	0.0027±<0.0001	0.0027±0.0001	0.399	0.662	0.900
**IVRT (sec)**	0.0194±0.0004	0.0199±0.0006	0.0185±0.0009	0.0202±0.0008	0.0207±0.0009	0.0223±0.0008	0.018	0.051	0.703
**FS (%)**	49.6±1.7	47.7±1.8	52.7±3.7	49.0±2.1	51.7±3.9	55.3±2.0	0.204	0.766	0.376
**Myocardial collagen and triglyceride; myocyte area**									
**HP (ug/mg wet wt)**	0.654±0.077	0.547±0.089	0.492±0.059	0.521±0.065	0.579±0.066	0.630±0.068	0.305	0.874	0.491
**Collagen (%)**	0.071±0.005	0.068±0.005	0.066±0.004	0.089±0.015	0.067±0.005	0.064±0.004	0.357	0.386	N/A
**TG (nM/g wet wt)**	62.98±14.25	48.56±10.86	37.81±6.56	22.46±1.99	28.37±5.87	33.84±6.69	0.019	0.355	0.268
**CSA (microns^2^)**	499.77±25.84	517.13±19.52	559.12±29.72	591.52±28.53	544.19±20.73	555.49±31.70	0.046	0.349	0.919

Data displayed as mean ± SE relevant to each treatment group (diet/strain). The p-values derived from 2-way ANOVA (representing diet, strain and interaction effects) are provided. LVW, left ventricular wall; LVID, left ventricular internal diameter; IVRT, isovolumic relaxation time; FS, fractional shortening; cr, cranial; ca, caudal; s, systole; d, diastole; CON, control; WES, Western; WES+DHA, Western + DHA; HP, hydroxyproline (n = 8–10); TG, triglyceride (n = 5–7); CSA, cross sectional area.

#### Protein oxidation

Carbonylation of LV protein was not different according to dietary treatment (p = 0.889; data not shown).

#### Myocardial adiponectin in Wistar rats

**Figure 4:**
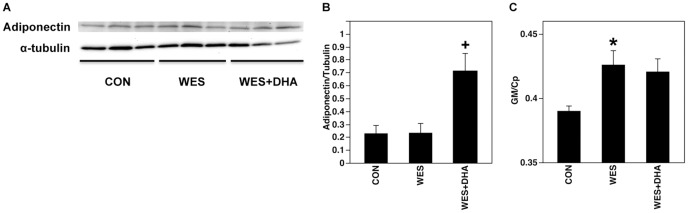
Adiponectin protein (A,B) and gene (C) expression in LV tissue of Wistar rats. Representative immunoblot (A) and graph of adiponectin/tubulin densitometry data (B). Adiponectin protein expression was increased in rats fed the WES+DHA diet compared to those fed the CON (p = 0.003) and WES (p = 0.004) diets (+p<0.01). Relative levels of *Adipoq* determined by RT-PCR (C). Data is expressed relative to the geometric mean (GM) of housekeeping genes *Rn18s* and *Gapdh*. Expression of *Adipoq* was increased by approximately 10% in WES fed rats compared to CON rats (p = 0.020) (*p<0.05).

Adiponectin protein expression was increased in rats fed the WES+DHA diet compared to those fed the CON and WES diets (Figure 4A,B).

Real-time PCR analysis revealed that expression of *Adipoq* was approximately 10% higher in WES fed rats compared to CON rats (p = 0.020). There was a trend toward increased *Adipoq* expression in WES+DHA fed rats compared to CON (p = 0.055) (Figure 4C).

#### Myocardial collagen and triglyceride; myocyte area

**Figure 5:**
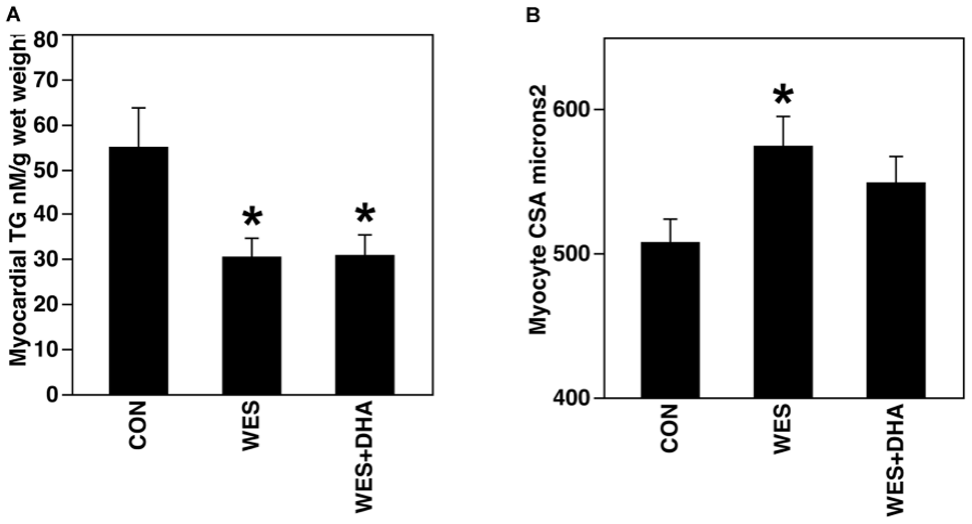
Myocardial TG content (A) and myocyte CSA (B). A: Rats fed the WES and WES+DHA diets had less myocardial TG compared to CON rats. B: Consumption of the WES diet was associated with greater myocyte CSA, compared to CON rats (*p<0.05).

Complete tabular data are contained within Tables 3 and S7. Results of the hydroxyproline assay (n = 8–10 due to tissue availability) and Masson's trichrome staining both support a lack of diet effect on myocardial collagen (Table S7). Compared to CON rats, those fed the WES and WES+DHA diets had less myocardial TG (n = 5–7 after exclusion of 2 outliers) (Figure 5A). Myocyte CSA was greater in rats fed the WES diet when compared to CON animals (Figure 5B).

#### Transmission electron microscopy

**Figure 6:**
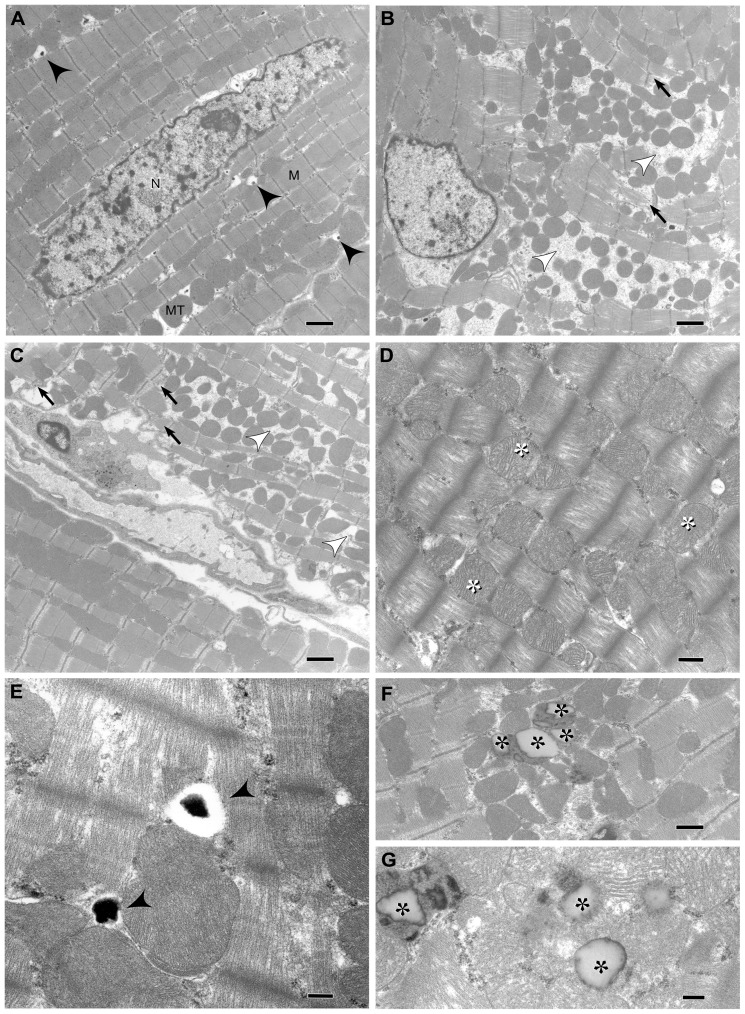
Electron micrographs of LV myocardium from Wistar rats (n = 2). CON (A) and WES and WES+DHA rats (B–G). Myocardium from CON rats showing nuclei (N), myofibers (M) and mitochondria (MT) with normal morphological features (A). Sarcomere periodicity and mitochondrial distribution between myofibers are normal. Occasional vacuoles with remnant matrix material are seen (black arrowheads), and appear to be associated with mitochondria. Left ventricle from WES (B) and WES+DHA (C) rats display multifocal loss of sarcomeres characterized by myofilament rarefaction and Z-line degeneration (black arrows). In areas of complete myofiber loss, there is coalescence of irregularly shaped, shrunken mitochondria (white arrowheads) (B and C). Some regions of myofilament rarefaction also contain mitochondria with swollen and/or disorganized cristae (white asterisks) (D). In WES and WES+DHA rats, there are greater numbers of mitochondria-associated vacuoles compared to CON rats, containing varying amounts of remnant matrix material, as shown in LV from a WES rat (black arrowheads) (E). Observed more frequently in WES+DHA rats than in WES rats are focal regions of lipid accumulation (*) that appear to be associated with, or contained within, degenerative mitochondria (F and G). Scale bars A–C: 1 µm; D, F: 0.5 µm; E, G: 0.25 µm.

Representative electron micrographs are presented in Figure 6. There were no consistent differences in nuclear morphology among treatment groups. Compared to myocardium from CON rats (Figure 6A), LV tissue from WES and WES+DHA rats contained degenerative myofibrils with small, coalescing mitochondria (Figure 6B,C). Also observed in areas of myofibril degeneration were mitochondria with swollen or disorganized cristae (Figure 6D). Mitochondria-associated vacuolation with varying amounts of degenerate matrix material were observed in all groups; however, the number of vacuoles and amount of remnant material was greatest in WES and WES+DHA rats (Figure 6A, E). Discrete lipid aggregates were observed in WES and WES+DHA rats, but this lesion was more commonly viewed in the latter group. In contrast to displacement of normal structures that one would anticipate with accumulation of exogenous lipid (e.g. steatosis), we observed that lipid aggregation appeared to be contained within, or closely associated with, degenerative mitochondria and thus were interpreted as representing fatty change.

### Strain effects

Averaged over diet, SD rats had higher terminal body weights (602±12 g) and weight gain (399±11 g) compared to Wistar rats (562±9 g, p = 0.012; 364±8 g, p = 0.015, respectively). Feed efficiency was also higher in SD rats (4.64±0.09) compared to Wistars (4.30±0.08; p = 0.008). Absolute heart and LV weights were greater in SD rats, but when indexed to body weight these differences were no longer present. Heart rates were higher in Wistar (407±8 bpm) than in SD rats (374±7 bpm; p = 0.005) (n = 4–9). There was an interaction effect on SBP such that consumption of the WES diet was associated with increased SBP in Wistar rats, but decreased SBP in SD rats (n = 4–9). Serum adiponectin was increased in Wistar (25.20±1.59 ug/mL) compared to SD (19.09±1.40 ug/mL; p = 0.002) rats. An effect of strain on the myocardial fatty acid profile was evident in that SD rats had lower concentrations of EPA (20∶5) and docosapentaenoic acid (DPA, 22∶5), and higher concentrations of arachidonic acid (AA, 20∶4). There was an interaction effect on EPA, DPA and tricosanoic acid concentrations. Regarding LV wall thickness, LVW_cr/s_ and LVW_ca/d_ were greater in SD rats (0.365±0.008 cm and 0.193±0.004 cm, respectively) compared to Wistar rats (0.339±0.007 cm and 0.179±0.004 cm, respectively). Similar to measured LV weight, LV mass calculated using echocardiographic data was greater in SD rats, but indexed masses were similar to those measured in Wistars. Regarding functional indices, there was a trend toward increased IVRT in WIS (0.0208±0.0005 sec) compared to SD rats (0.0196±0.0005; p = 0.051).

## Discussion

We propose that defining alterations in cardiac structure and function attributable to diet may be best accomplished by investigating the effects of combined fatty acid moieties as a relevant diet, consumed in the *in vivo* setting of intact anti-inflammatory and antioxidant systems. Accordingly, the aim of this study was to determine whether LVH occurs in association with intake of a Western diet, in the absence of hypertension or apparent insulin resistance. We also sought to identify whether concomitant supplementation with dietary DHA attenuated any hypertrophic response, and whether strain differences existed. This study revealed diet-specific changes in serum lipids, serum and myocardial adiponectin, myocardial fatty acid composition, LV thickening and diastolic function, myocardial TG and myocyte hypertrophy. Interestingly, despite increased circulating and myocardial adiponectin in WES+DHA rats, LV thickening and myocardial dysfunction were more pronounced in this group compared with rats fed the WES diet alone. In the absence of collagen or lipid accretion, myocyte hypertrophy appears to be the most likely contributor to gross LV thickening. Myocyte injury and disrupted contractile and mitochondrial structure observed with TEM provide ultrastructural evidence of lesions consistent with dysfunction and increased myocyte CSA. There was a strain effect on the myocardial fatty acid profile and serum adiponectin concentration; these differences may partly underlie the varied and sometimes contradictory results of rodent dietary obese studies.

### Morphometric, hemodynamic and metabolic profiles

Dietary treatment was not associated with changes in feed efficiency, weight gain or terminal visceral adipose mass. These findings are consistent with those from an 8-week feeding study examining metabolic responses in lean and obese Zucker rats; obese, but not lean, rats demonstrated altered energy expenditure and adipose mass in response to dietary fat content or type.[34] In addition, SBP, HR, glucose, insulin and calculated HOMA were unchanged by dietary treatment. Collectively, these findings suggest that primary outcomes may be interpreted in the absence of increased body mass, adiposity, afterload and insulin resistance.

Unexpectedly, whereas WES rats displayed regional thickening of the cranial LV wall, WES + DHA rats had global LV thickening (i.e. both cranial and caudal walls). Circulating adiponectin is inversely related to LV mass in humans,[35] and rodent studies indicate that adiponectin may afford protection from myocardial hypertrophy attributable to high-fat feeding [36] and oxidative stress.[37] Evidence suggests that antihypertrophic effects may partly be mediated by endogenous myocardial adiponectin acting in an autocrine fashion on AdipoR1 and AdipoR2 receptors.[36], [38] We hypothesized that reduced serum and/or myocardial adiponectin may be involved in the diet-induced LVH changes; however, in the present study, serum and myocardial adiponectin were increased in rats fed DHA. It is possible that the more diffuse LVH observed in WES+DHA rats may be partly attributed to myocardial adiponectin resistance. Resistance to adiponectin has been demonstrated in diabetic and insulin resistant rodents fed high fat diets.[39], [40] Future work will examine this possibility. It would also appear that protein oxidation is not a major factor in cardiac tissue adaptation to diet.

Both high-fat diets were associated with reduced serum TG and FFA, and serum TG were lowered further in WES+DHA compared to WES rats. Others have demonstrated similar or reduced serum TG and FFA in Wistar rats after 4 and 20 weeks of high-fat feeding compared to control animals fed a low-fat diet.[41] A clear serum TG- and FFA-lowering effect has been demonstrated in rats fed a high-fat diet enriched with fish oil (EPA and DHA).[42]

### Myocardial structure and function

Effects of dietary fats on myocardial structure and function in the setting of pressure overload have been demonstrated.[43]–[45] Less is known about the role of dietary fatty acid composition in the absence of concomitant hypertension, myocardial ischemia, diabetes or obesity. In the present study, both fat-fed groups exhibited echocardiographic evidence of increased LV thickness and reduced LV chamber diameter, consistent with concentric hypertrophy. Heart and LV masses were not different among treatment groups. Similar findings were observed in mice overexpressing diacylglycerol acyl transferase, the enzyme that promotes TG synthesis; some subjects developed myocyte hypertrophy and myocardial collagen and lipid accrual in the absence of increased organ weight.[46] In the present study, this phenomenon may be due to relatively less myocardial TG in the fat fed animals, to focal thickening that does not contribute to overall mass or to insufficient sample size for detection of this change. Compared to the regional LV thickening observed in WES rats, the more global LV thickening observed in WES + DHA rats suggests that this early and prognostic structural change is not attenuated by DHA supplementation. Other work supports these findings; fish oil supplementation did not attenuate hypertrophy in Wistar rats subjected to aortic banding.[45] Importantly, however, this same study revealed that despite unchanged structure, *ex vivo* myocardial function was in fact improved by fish oil consumption. It is possible that more sensitive measures of function (i.e. isolated heart or myocyte preparations) or echocardiographic assessment under conditions of myocardial stress (i.e. dobutamine administration) may have identified levels of systolic dysfunction that were not detected in the present study. One must further consider that gross hypertrophy can be adaptive, and evidence suggests that this phenotype may be partly determined by the fatty acid milieu.[47], [48] Future studies of myocardial gene and protein expression relevant to adaptive versus maladaptive LVH may identify unique profiles in WES compared to WES+DHA rats. Regarding diastolic function, similar to healthy overweight and obese humans,[3], [4] WES+DHA rats had prolonged IVRT compared to CON and WES rats. Comparing the collective data from these experiments to other rodent studies, echocardiography revealed no change in myocardial structure and function in response to 8 weeks of high saturated fat and PUFA feeding,[49] while others observed increased LV mass and impaired contractile function in mice fed a high fat diet for 20 weeks.[50] Taken together, the echocardiographic alterations observed in these WES+DHA fed rats reflect changes observed in obese humans; that is, LV thickening and diastolic dysfunction with preserved or augmented systolic function.[51], [52]

### Myocardial fatty acid composition

Dietary fatty acids determine the fatty acid composition of the myocardium, and changes in myocellular lipids are associated with altered intracellular signaling, including pathways that may be important in modulating myocyte metabolism, hypertrophy, contractile function and ultimately survival.[53]–[57] Dietary DHA appears to be readily incorporated into tissue membranes, and specifically myocardial phospholipids, regardless of concomitant n-6 feeding.[58] Our data are consistent with this idea, as WES + DHA rats accumulated myocardial DHA despite incorporation into a Western diet high in absolute n-6, though it should be noted that the n6∶n3 was low. Interestingly, though many rodent studies suggest a lack of toxicity with high DHA intake,[59] others have shown that rats consuming DHA at levels at or above those used in the current study had lower levels of myocardial phospholipid DHA.[24]–[26] The reasons for this are not clear, but may be related to base diet composition as the aforementioned studies incorporated control, rather than Western, diets. Accumulation of ALA and DHA in CON rats that consumed relatively high amounts of ALA is not surprising, especially in light of evidence suggesting that ALA is readily incorporated into tissue membranes in the absence of LA (i.e. when the n6∶n3 is low).[60]

### Myocyte cross sectional area

Results of this study support the idea that increased myocyte CSA is the most likely contributor to gross LV hypertrophy. Rats fed the WES diet had the greatest increase in mean cell size compared to CON rats. There was a trend toward increased myocyte size in WES+DHA rats compared to CON (p = 0.123); thus, DHA supplementation may not fully attenuate the increased cell size associated with WES feeding. However, WES+DHA rats had more global LV hypertrophy. Given that WES+DHA rats did not show evidence of collagen or lipid accumulation, there is a possibility that the regions selected for myocyte measurement did not wholly reflect (i.e. underrepresented) the LV. There is substantial evidence that diet modulates myocyte hypertrophy attributed to alternative primary etiologies such as hypertension [61] and altered lipid metabolism.[62] Less is known about myocyte hypertrophy in response to diet alone, in the absence of overt comorbidities, and in the *in vivo* milieu of intact compensatory/counter mechanisms. The findings of this study are consistent with those of others in that a Western diet appears to be associated with myocyte hypertrophy in healthy rodents,[63] and further support the idea that DHA supplementation does not significantly alter this outcome.

The presence of myocyte hypertrophy in the present rodent study is consistent with findings in obese humans. Myocyte hypertrophy was the principal finding in hearts of obese individuals without premortem evidence of heart disease.[8] Hypertrophic myocytes were also present in obese individuals that died of an array of cardiovascular and noncardiovascular causes.[2] Hypertrophic stimuli, and subsequent genotypic and phenotypic responses, are very diverse.[64], [65] Certainly a measure of CSA only defines the presence of the phenomenon, and it is likely that myocyte gene expression, signaling pathways and subsequent preservation or deterioration of structure and function are different according to fatty acid milieu,[48] degree of adiposity, adipokine profile [66] and metabolic aberrancy. Future work will explore these possibilities.

### Myocardial lipid accumulation

The results of this study indicate that myocardial TG accumulation does not contribute to gross LV thickening that occurred in association with the diets tested. There is disparate evidence regarding the occurrence and impact of myocardial TG (i.e. neutral lipid) accumulation in obesity. Evidence suggests that overweight and obese individuals have increased myocardial TG deposition compared to lean subjects.[67], [68] In contrast, LV tissue from humans with end-stage nonischemic heart failure revealed no difference in intramyocardial lipid staining in hearts from lean and obese subjects.[68] Further, postmortem examination of 12 obese individuals without evidence of hypertension or myocardial ischemia identified only scant fatty infiltration in 3 of the subjects.[8] Rodent high-fat feeding studies reveal both increased [69]–[71] and unchanged [72] myocardial TG. Reduced myocardial TG in the fat fed rats observed in the present study is consistent with reduced serum FFA and TG observed in these groups.

### Transmission electron microscopy

At an ultrastructural level, vacuolated mitochondria with swollen cristae, disorganized sarcomeres resulting in disruption of integrity and loss of myofibers, and accumulation of lipids have been documented in fat-fed mice, hypertensive rats, and genetically obese rats.[61], [63], [71], [73]–[75] Similar myocardial lesions have been reported concomitant with dyslipidemia, hyperglycemia/insulinemia and/or myocardial TG accumulation in normal rodents as a consequence of dietary manipulations.[73], [74]. In the present study, WES diet consumption was associated with degeneration of the contractile apparatus and altered mitochondrial structure, without manifest systemic functional dysregulation. The lipid deposition observed in myocytes observed in our study is very likely a sequel of degeneration of cellular organelles leading to aggregation of membrane lipids, and not a consequence of accumulation of circulating TG since myocardial TG content was reduced in both fat-fed groups. Furthermore, no contortion of cellular organelles was observed with TEM; if the lipid deposits were due to accumulation of exogenous TG within the cell, typically displacement of cellular organelles would be expected. Thus this process of lipid aggregation, termed “fatty change”, reflects reversible injury and may be accompanied by cell swelling. Although it was not feasible to obtain stringent quantitative data from the small number of tissue samples subjected to TEM, our qualitative evaluation nonetheless suggests that DHA supplementation does not prevent the occurrence of the ultrastructural lesions.

### Strain considerations

When possible, the above discussion has focused on models of OC without concomitant genetic anomalies or induced pathology (i.e. aortic banding, spontaneous hypertension). Within these studies, the data collectively suggest that Wistar and SD rats have distinct lipid metabolism,[20] and Wistars may develop myocardial pathology with shorter dietary interventions.[70], [76] Additional strain differences in metabolic and myocardial responses to high-fat feeding have been demonstrated.[77] Our findings provide evidence for a strain effect on feed efficiency, body weight, heart rate and serum adiponectin and the myocardial FA profile. Regarding the latter, the SD myocardial phospholipid fraction was more enriched in the n-6 AA, while myocardium from Wistar rats contained more total n-3 fatty acids. Differences in fatty acid incorporation should be considered when designing studies of related outcomes. The interaction effect on SBP suggests that the hemodynamic response to dietary treatment is different in SD and Wistar rats. Increased SBP in Wistar rats in response to WES feeding may partly underlie observations made by others; namely, the development of myocardial hypertrophy and impaired function with just 16 weeks of high-fat, high-simple carbohydrate feeding.[23], [27]

### Limitations

Study limitations include the use of interventricular septal tissue for measurement of myocardial fatty acids, hydroxyproline and TG, given that echocardiographic measurements of structure and function were derived from images of the cranial and caudal LV free walls.

### Conclusions

The results of this study support the idea that diet alone, in the absence of hypertension or overt insulin resistance, is associated with changes in myocardial structure and function that are relevant to human OC. Consumption of a Western diet was associated with LV thickening in rats, most likely attributed to increased myocyte size. It is widely appreciated that dietary n-3 PUFA are protective against cardiomyopathy, yet supplementation of the Western diet with DHA was associated with more global LV thickening and development of diastolic dysfunction, despite increased circulating and myocardial adiponectin and reduced serum TG. Disruption of normal contractile and mitochondrial structure may underlie gross changes, with reversible injury and associated cell swelling possibly contributing to increased myocyte size. It is possible that these outcomes reflect a robust accumulation of DHA in the myocardial phospholipid fraction. Two strains commonly used in dietary obesity studies displayed distinct myocardial fatty acid profiles and serum adiponectin concentrations. These strain effects may have important effects on cardiac outcomes, and may contribute to inconsistent data generated from use of the dietary obese model.

## Supporting Information

Table S1 Fatty acid composition of diets (% of total diet fatty acids).
(DOCX)

Table S2 Primer pair sequences for the *Adipoq*, *Rn18s* and *Gapdh* genes.
(DOCX)

Table S3 Initial body weights, energy intake and efficiency, absolute tissue masses and selected serum metabolic indices, according to diet and strain.
(DOCX)

Table S4 Fatty acid profile of myocardial phospholipid fractions according to diet and strain.
(DOCX)

Table S5 Data summary including diet consumption, body morphometry, tissue masses, hemodynamics and serum metabolic indices, according to diet.
(DOCX)

Table S6 Fatty acid profile of myocardial phospholipid fractions according to diet.
(DOCX)

Table S7 Summary of echocardiographic measurements, myocardial hydroxyproline, collagen and triglyceride content and myocyte cross sectional area, according to diet.
(DOCX)
