# Supplementary material for: Docosahexaenoic Acid Supplementation Does Not Improve Western Diet-Induced Cardiomyopathy in Rats
Source: PLoS One. 2012 Dec 26;7(12):e51994. doi: 10.1371/journal.pone.0051994 (PMC3530602; doi:10.1371/journal.pone.0051994)
Supplement: Table S1 — Fatty acid composition of diets (% of total diet fatty acids). (DOCX) [file pone.0051994.s001.docx]

**Table S1** Fatty acid composition of diets (% of total diet fatty acids).

|  | **CON** | **WES** | **WES+DHA** |
| --- | --- | --- | --- |
| **8:0** | 2.5 | 3.8 | 3.6 |
| **10:0** | 2.0 | 3.0 | 3.0 |
| **12:0** | 12.5 | 19.4 | 18.9 |
| **14:0** | 7.0 | 10.6 | 11.5 |
| **16:0** | 13.8 | 17.3 | 17.2 |
| **18:0** | 8.9 | 11.4 | 10.7 |
| **18:1 n-9** | 19.3 | 19.0 | 19.4 |
| **18:2 n-6 (LA)** | 16.3 | 9.9 | 5.8 |
| **18:3 n-3 (ALA)** | 14.3 | 0.5 | 0.5 |
| **22:6 n-3 (DHA)** | 0.0 | 0.0 | 4.4 |
| **n-6** | 0.67 | 2.9 | 1.7 |
| **n-3** | 0.58 | 0.14 | 1.4 |
| **n-6: n-3** | 1.2 | 21.0 | 1.2 |

CON, control; WES, Western; WES+DHA, Western + DHA. n-3, omega-3

polyunsaturated fatty acid; n-6, omega-6 polyunsaturated fatty acid; LA, linoleic acid; ALA, α-linolenic acid; DHA, docosahexaenoic acid.
