# Supplementary material for: Docosahexaenoic Acid Supplementation Does Not Improve Western Diet-Induced Cardiomyopathy in Rats
Source: PLoS One. 2012 Dec 26;7(12):e51994. doi: 10.1371/journal.pone.0051994 (PMC3530602; doi:10.1371/journal.pone.0051994)
Supplement: Table S2 — Primer pair sequences for the Adipoq, Rn18s and Gapdh genes. (DOCX) [file pone.0051994.s002.docx]

**Table S2** Primer pair sequences for the *Adipoq*, *Rn18s* and *Gapdh* genes.

| Adipoq Forward | CTGTTGCAAGCGCTCCTGTT |
| --- | --- |
| Adipoq Reverse | CCGGTATCCCATTGTGACCA |
| Rn18s Forward | GAGGCCCTGTAATTGGAATGAG |
| Rn18s Reverse | GCAGCAACTTTAATATACGCTATTGG |
| Gapdh Forward | CCAGGGCTGCCTTCTCTTGT |
| Gapdh Reverse | TGATGGGTTTCCCGTTGATG |
