# Supplementary material for: Docosahexaenoic Acid Supplementation Does Not Improve Western Diet-Induced Cardiomyopathy in Rats
Source: PLoS One. 2012 Dec 26;7(12):e51994. doi: 10.1371/journal.pone.0051994 (PMC3530602; doi:10.1371/journal.pone.0051994)
Supplement: Table S3 — Initial body weights, energy intake and efficiency, absolute tissue masses and selected serum metabolic indices, according to diet and strain. (DOCX) [file pone.0051994.s003.docx]

**Table S3** Initial body weights, energy intake and efficiency, absolute tissue masses and selected serum metabolic indices, according to diet and strain.

|  | **CON** | | **WES** | | **WES + DHA** | | **p value (diet)** | **p value (strain)** | | **p value (int)** |
| --- | --- | --- | --- | --- | --- | --- | --- | --- | --- | --- |
|  | **SD** | **WIS** | **SD** | **WIS** | **SD** | **WIS** |  |  | |  |
| **Initial body weight, energy intake, efficiency and absolute tissue weights** | | | | | | | | | | |
| **Initial body weight (g)** | 198 ± 6 | 198 ± 6 | 205 ± 4 | 198 ± 5 | 206 ± 5 | 197 ± 7 | 0.737 | 0.258 | | 0.695 |
| **Total kcal consumed** | 8502 ± 180 | 8215 ± 157 | 8757 ± 312 | 8570 ± 116 | 8552 ± 255 | 8617 ± 139 | 0.313 | 0.419 | | 0.678 |
| **Feed efficiency** | 4.85 ± 0.13 | 4.29 ± 0.13 | 4.41 ± 0.20 | 4.27 ± 0.18 | 4.65 ± 0.11 | 4.34 ± 0.14 | 0.290 | 0.008 | | 0.382 |
| **Visceral adipose wt (g)** | 6.76 ± 0.81 | 5.06 ± 0.40 | 5.80 ± 0.65 | 5.17 ± 0.44 | 5.83 ± 0.53 | 5.56 ± 0.62 | 0.775 | 0.076 | | 0.447 |
| **Heart wt (g)** | 1.45 ± 0.04 | 1.21 ± 0.05 | 1.30 ± 0.05 | 1.23 ± 0.03 | 1.37 ± 0.05 | 1.27 ± 0.03 | 0.338 | <0.001 | | 0.125 |
| **LV wt (g)** | 0.707 ± 0.018 | 0.644 ± 0.023 | 0.659 ± 0.018 | 0.634 ± 0.023 | 0.717 ± 0.030 | 0.665 ± 0.022 | 0.164 | 0.017 | | 0.706 |
| **Serum measurements and HOMA** | | | | | | | | | | |
| **Leptin (ng/mL)** | 12.4 ± 3.38 | 6.68 ± 0.94 | 9.29 ± 2.44 | 6.53 ± 1.26 | 7.48 ± 2.03 | 7.52 ± 1.49 | 0.538 | 0.430 | N/A | |
| **Insulin (ng/mL)** | 2.53 ± 0.47 | 2.16 ± 0.25 | 2.59 ± 0.33 | 2.48 ± 0.34 | 2.77 ± 0.44 | 3.06 ± 0.31 | 0.303 | 0.830 | 0.673 | |
| **Glucose (mg/dl)** | 191.3 ± 11.8 | 183.6 ± 6.3 | 194.1 ± 10.3 | 180.4 ± 9.9 | 179.6 ± 7.1 | 170.8 ± 4.1 | 0.295 | 0.167 | 0.936 | |
| **HOMA** | 30.47 ± 6.24 | 23.71 ± 2.68 | 31.12 ± 4.51 | 27.91 ± 4.32 | 31.17 ± 6.12 | 31.86 ± 3.63 | 0.657 | 0.433 | 0.743 | |

Data displayed as mean ± SE relevant to each treatment group (diet/strain). The p-values derived from 2-way ANOVA (representing diet, strain and interaction effects) are provided. CON, control; WES, Western; WES+DHA, Western + DHA.
