# Supplementary material for: Docosahexaenoic Acid Supplementation Does Not Improve Western Diet-Induced Cardiomyopathy in Rats
Source: PLoS One. 2012 Dec 26;7(12):e51994. doi: 10.1371/journal.pone.0051994 (PMC3530602; doi:10.1371/journal.pone.0051994)
Supplement: Table S4 — Fatty acid profile of myocardial phospholipid fractions according to diet and strain. (DOCX) [file pone.0051994.s004.docx]

**Table S4** Fatty acid profile of myocardial phospholipid fractions according to diet and strain.

|  | **CON** | | **WES** | | **WES + DHA** | | **p value (diet)** | **p value (strain)** | **p value (int)** |
| --- | --- | --- | --- | --- | --- | --- | --- | --- | --- |
|  | **SD** | **WIS** | **SD** | **WIS** | **SD** | **WIS** |  |  |  |
| **16:0** | 10.7 ± 0.387 | 10.3 ± 0.149 | 8.88 ± 0.138 | 8.52 ± 0.173 | 10.4 ± 0.056 | 10.3 ± 0.113 | < 0.0001 | 0.100 | 0.838 |
| **18:0** | 21.4 ± 0.225 | 20.7 ± 0.079 | 23.9 ± 0.319 | 24.2 ± 0.220 | 22.7 ± 0.238 | 23.1 ± 0.374 | < 0.0001 | 0.944 | 0.114 |
| **18:1 n-7** | 4.56 ± 0.094 | 4.72 ± 0.131 | 2.86 ± 0.143 | 2.96 ± 0.132 | 2.91 ± 0.067 | 2.53 ± 0.072 | <0.0001 | 0.689 | 0.051 |
| **18:1 n-9** | 4.49 ± 0.116 | 4.72 ± 0.155 | 3.17 ± 0.198 | 3.22 ± 0.073 | 3.55 ± 0.117 | 3.31 ± 0.078 | < 0.0001 | 0.913 | 0.221 |
| **18:2 n-6 (LA)** | 16.0 ± 0.948 | 18.4 ± 0.683 | 13.3 ± 0.661 | 12.9 ± 0.950 | 12.3 ± 0.313 | 12.6 ± 0.189 | < 0.0001 | 0.178 | 0.148 |
| **18:3 n-3 (ALA)** | 0.456 ± 0.017 | 0.483 ± 0.011 | 0.160 ± 0.034 | 0.151 ± 0.010 | 0.132 ± 0.032 | 0.129  ± 0.025 | < 0.0001 | 0.797 | 0.725 |
| **20:4 n-6 (AA)** | 20.3 ± 0.643 | 18.2 ± 0.535 | 24.9 ± 0.233 | 24.6 ± 0.228 | 10.5 ± 0.346 | 10.1 ± 0.471 | < 0.0001 | 0.020 | 0.095 |
| **20:5** | 0.540 ± 0.048 | 0.898 ± 0.020 | 0±0 | 0±0 | 0.387 ± 0.077 | 0.755 ± 0.028 | < 0.0001 | < 0.0001 | 0.0002 |
| **22:5 n-3** | 1.69 ± 0.502 | 3.34 ± 0.105 | 1.65 ± 0.114 | 2.23 ± 0.091 | 0.771 ± 0.044 | 1.14 ± 0.027 | < 0.0001 | 0.0001 | 0.020 |
| **22:6 n-3 (DHA)** | 14.4 ± 0.742 | 13.2 ± 0.560 | 10.9 ± 0.771 | 10.7 ± 0.528 | 31.9 ± 0.692 | 31.4 ± 0.802 | < 0.0001 | 0.298 | 0.756 |
| **23:0** | 0.261 ± 0.005 | 0.259 ± 0.024 | 1.32 ± 0.074 | 1.52 ± 0.050 | 0±0 | 0±0 | < 0.0001 | 0.04 | 0.02 |
| **23:1** | 0.201 ± 0.028 | 0.127 ± 0.006 | 4.43 ± 0.691 | 4.22 ± 0.335 | 0±0 | 0±0 | < 0.0001 | 0.72 | 0.95 |

Data displayed as mean area% ± SE relevant to each treatment group (diet/strain); n = 4. The p-values derived from 2-way ANOVA (representing diet, strain and interaction effects) are provided. CON, control; WES, Western; WES+DHA, Western + DHA. n-3, omega-3 polyunsaturated fatty acid; n-6, omega-6 polyunsaturated fatty acid; LA, linoleic acid; ALA, α-linolenic acid; AA, arachidonic acid; DHA, docosahexaenoic acid.
