# Supplementary material for: Docosahexaenoic Acid Supplementation Does Not Improve Western Diet-Induced Cardiomyopathy in Rats
Source: PLoS One. 2012 Dec 26;7(12):e51994. doi: 10.1371/journal.pone.0051994 (PMC3530602; doi:10.1371/journal.pone.0051994)
Supplement: Table S5 — Data summary including diet consumption, body morphometry, tissue masses, hemodynamics and serum metabolic indices, according to diet. (DOCX) [file pone.0051994.s005.docx]

**Table S5** Data summary including diet consumption, body morphometry, tissue masses, hemodynamics and serum metabolic indices, according to diet.

|  | **CON** | **WES** | **WES+DHA** | **p** |
| --- | --- | --- | --- | --- |
| **Diet consumption, body morphometry, tissue weights** | | | |  |
| **Total kcal consumed** | 8359 ± 121 | 8663 ± 163 | 8584 ± 142 | 0.313 |
| **Feed efficiency** | 4.57 ± 0.11 | 4.34 ± 0.13 | 4.50 ± 0.09 | 0.290 |
| **Initial body weight (g)** | 198 ± 4 | 201 ± 3 | 202 ± 4 | 0.737 |
| **Body wt (g)** | 581 ± 14 | 577 ± 15 | 588 ± 13 | 0.842 |
| **Wt gain (g)** | 383 ± 12 | 376 ± 14 | 386 ± 11 | 0.814 |
| **Visceral adipose wt (g)** | 5.91 ± 0.48 | 5.47 ± 0.38 | 5.69 ± 0.40 | 0.775 |
| **Visceral adipose: body wt** | 0.0100 ± 0.0006 | 0.0096 ± 0.0006 | 0.0096 ± 0.0006 | 0.873 |
| **Heart wt (g)** | 1.33 ± 0.04 | 1.27 ± 0.03 | 1.32 ± 0.03 | 0.338 |
| **Heart: body wt** | 0.0023 ± 4.3152e^-5^ | 0.0023 ± 3.6183e^-5^ | 0.0023 ± 3.8113e^-5^ | 0.581 |
| **LV wt (g)** | 0.677 ± 0.016 | 0.646 ± 0.015 | 0.691 ± 0.019 | 0.164 |
| **LV:body wt** | 0.0012 ± 3.0472e^-5^ | 0.0011 ± 2.7651e^-5^ | 0.0012 ± 2.6474e^-5^ | 0.582 |
| **Heart rate and blood pressure** | | | |  |
| **HR (bpm)** | 385 ± 7.74 | 398 ± 13.1 | 383 ± 9.62 | 0.511 |
| **SBP (mm Hg)** | 146.15 ± 7.42 | 146.9 ± 11.35 | 139.4 ± 3.49 | 0.750 |
| **Serum measurements and HOMA** | | | |  |
| **Adiponectin (ug/mL)** | 19.41 ± 1.25 | 19.53 ± 1.33 | 27.31 ± 2.53 | 0.001 |
| **FFA (uEq/L)** | 709.3 ± 33.1 | 593.6 ± 24.7 | 570.4 ± 22.2 | 0.002 |
| **Glucose (mg/dl)** | 187.5 ± 6.6 | 187.3 ± 7.1 | 175.4 ± 4.2 | 0.295 |
| **HOMA** | 27.09 ± 3.40 | 29.51 ± 3.06 | 31.50 ± 3.55 | 0.657 |
| **Insulin (ng/mL)** | 2.35 ± 0.26 | 2.54 ± 0.23 | 2.91 ± 0.27 | 0.303 |
| **Leptin (ng/mL)** | 9.5 ± 1.83 | 7.9 ± 1.38 | 7.5 ± 1.25 | 0.538 |
| **TG (mg/dl)** | 106.4 ± 11.7 | 76.4 ± 7.8 | 54.1 ± 3.8 | < 0.001 |

Data displayed as mean ± SE. The p-values relevant to diet effect are derived from 2-way ANOVA. CON, control; WES, Western; WES+DHA, Western + DHA. HR/SBP n = 4-9.
