# Supplementary material for: Docosahexaenoic Acid Supplementation Does Not Improve Western Diet-Induced Cardiomyopathy in Rats
Source: PLoS One. 2012 Dec 26;7(12):e51994. doi: 10.1371/journal.pone.0051994 (PMC3530602; doi:10.1371/journal.pone.0051994)
Supplement: Table S6 — Fatty acid profile of myocardial phospholipid fractions according to diet. (DOCX) [file pone.0051994.s006.docx]

**Table S6** Fatty acid profile of myocardial phospholipid fractions according to diet.

|  | **CON** | **WES** | **WES+DHA** | **p** |
| --- | --- | --- | --- | --- |
| **16:0** | 10.5 ± 0.202 | 8.70 ± 0.123 | 10.3 ± 0.064 | < 0.0001 |
| **18:0** | 21.1 ± 0.164 | 24.1 ± 0.185 | 22.9 ± 0.221 | < 0.0001 |
| **18:1 n-7** | 4.6 ± 0.081 | 2.91 ± 0.092 | 2.72 ± 0.084 | <0.0001 |
| **18:1 n-9** | 4.6 ± 0.099 | 3.2 ± 0.098 | 3.4 ± 0.080 | < 0.0001 |
| **18:2 n-6 (LA)** | 17.2 ± 0.703 | 13.1 ± 0.540 | 12.5 ± 0.181 | < 0.0001 |
| **18:3 n-3 (ALA)** | 0.469 ± 0.011 | 0.155 ± 0.016 | 0.131 ± 0.019 | < 0.0001 |
| **20:4 n-6 (AA)** | 19.2 ± 0.552 | 24.7 ± 0.159 | 10.3 ± 0.281 | < 0.0001 |
| **20:5** | 0.719 ± 0.072 | 0 ± 0 | 0.571 ± 0.079 | < 0.0001 |
| **22:5 n-3** | 2.51 ± 0.391 | 1.94 ± 0.128 | 0.957 ± 0.074 | < 0.0001 |
| **22:6 n-3 (DHA)** | 13.8 ± 0.485 | 10.8 ± 0.434 | 31.6 ± 0.497 | < 0.0001 |
| **23:0** | 0.260 ± 0.012 | 1.42 ± 0.056 | 0 ± 0 | < 0.0001 |
| **23:1** | 0.164 ± 0.019 | 4.33 ± 0.358 | 0 ± 0 | < 0.0001 |

Data displayed as mean area% ± SE; n = 4. The p-values relevant to diet effect are derived from 2-way ANOVA. CON, control; WES, Western; WES+DHA, Western + DHA. n-3, omega-3 polyunsaturated fatty acid; n-6, omega-6 polyunsaturated fatty acid; LA, linoleic acid; ALA, α-linolenic acid; AA, arachidonic acid; DHA, docosahexaenoic acid.

**Table S2** Primer pair sequences for the *Adipoq*, *Rn18s* and *Gapdh* genes.

| Adipoq Forward | CTGTTGCAAGCGCTCCTGTT |
| --- | --- |
| Adipoq Reverse | CCGGTATCCCATTGTGACCA |
| Rn18s Forward | GAGGCCCTGTAATTGGAATGAG |
| Rn18s Reverse | GCAGCAACTTTAATATACGCTATTGG |
| Gapdh Forward | CCAGGGCTGCCTTCTCTTGT |
| Gapdh Reverse | TGATGGGTTTCCCGTTGATG |

**Table S3** Initial body weights, energy intake and efficiency, absolute tissue masses and selected serum metabolic indices.

|  | **CON** | | **WES** | | **WES + DHA** | | **p value (diet)** | **p value (strain)** | | **p value (int)** |
| --- | --- | --- | --- | --- | --- | --- | --- | --- | --- | --- |
|  | **SD** | **WIS** | **SD** | **WIS** | **SD** | **WIS** |  |  | |  |
| **Initial body weight, energy intake, efficiency and absolute tissue weights** | | | | | | | | | | |
| **Initial body weight (g)** | 198 ± 6 | 198 ± 6 | 205 ± 4 | 198 ± 5 | 206 ± 5 | 197 ± 7 | 0.737 | 0.258 | | 0.695 |
| **Total kcal consumed** | 8502 ± 180 | 8215 ± 157 | 8757 ± 312 | 8570 ± 116 | 8552 ± 255 | 8617 ± 139 | 0.313 | 0.419 | | 0.678 |
| **Feed efficiency** | 4.85 ± 0.13 | 4.29 ± 0.13 | 4.41 ± 0.20 | 4.27 ± 0.18 | 4.65 ± 0.11 | 4.34 ± 0.14 | 0.290 | 0.008 | | 0.382 |
| **Visceral adipose wt (g)** | 6.76±.81 | 5.06±.40 | 5.80±.65 | 5.17±.44 | 5.83±.53 | 5.56±.62 | 0.775 | 0.076 | | 0.447 |
| **Heart wt (g)** | 1.45±  .04 | 1.21±  .05 | 1.30±  .05 | 1.23±  .03 | 1.37±  .05 | 1.27±  .03 | 0.338 | <0.001 | | 0.125 |
| **LV wt (g)** | .707±  .018 | .644±  .023 | .659±  .018 | .634±  .023 | .717±  .030 | .665±  .022 | 0.164 | 0.017 | | 0.706 |
| **Serum measurements and HOMA** | | | | | | | | | | |
| **Leptin (ng/mL)** | 12.4±  3.38 | 6.68±  0.94 | 9.29±  2.44 | 6.53±  1.26 | 7.48±  2.03 | 7.52±  1.49 | 0.538 | 0.430 | N/A | |
| **Insulin (ng/mL)** | 2.53±  0.47 | 2.16±  0.25 | 2.59±  0.33 | 2.48±  0.34 | 2.77±  0.44 | 3.06±  0.31 | 0.303 | 0.830 | 0.673 | |
| **Glucose (mg/dl)** | 191.3±  11.8 | 183.6±  6.3 | 194.1±  10.3 | 180.4±  9.9 | 179.6±  7.1 | 170.8±  4.1 | 0.295 | 0.167 | 0.936 | |
| **HOMA** | 30.47±  6.24 | 23.71±  2.68 | 31.12±  4.51 | 27.91±  4.32 | 31.17±  6.12 | 31.86±  3.63 | 0.657 | 0.433 | 0.743 | |

Data displayed as mean ± SE relevant to each treatment group (diet/strain). The p-values derived from 2-way ANOVA (representing diet, strain and interaction effects) are provided. CON, control; WES, Western; WES+DHA, Western + DHA.

**Table S4** Fatty acid profile of myocardial phospholipid fractions.

|  | **CON** | | **WES** | | **WES + DHA** | | **p value (diet)** | **p value (strain)** | **p value (int)** |
| --- | --- | --- | --- | --- | --- | --- | --- | --- | --- |
|  | **SD** | **WIS** | **SD** | **WIS** | **SD** | **WIS** |  |  |  |
| **16:0** | 10.7±  .387 | 10.3±  .149 | 8.88±  .138 | 8.52±  .173 | 10.4±  .056 | 10.3±  .113 | < 0.0001 | 0.100 | 0.838 |
| **18:0** | 21.4±  .225 | 20.7±  .079 | 23.9±  .319 | 24.2±  .220 | 22.7±  .238 | 23.1±  .374 | < 0.0001 | 0.944 | 0.114 |
| **18:1 n-7** | 4.56±  .094 | 4.72±  .131 | 2.86±  .143 | 2.96±  .132 | 2.91±  .067 | 2.53±  .072 | <0.0001 | 0.689 | 0.051 |
| **18:1 n-9** | 4.49±  .116 | 4.72±  .155 | 3.17±  .198 | 3.22±  .073 | 3.55±  .117 | 3.31±  .078 | < 0.0001 | 0.913 | 0.221 |
| **18:2 n-6 (LA)** | 16.0±  .948 | 18.4±  .683 | 13.3±  .661 | 12.9±  .950 | 12.3±  .313 | 12.6±  .189 | < 0.0001 | 0.178 | 0.148 |
| **18:3 n-3 (ALA)** | .456±  .017 | .483±  .011 | .160±  .034 | .151±  .010 | .132±  .032 | .129±  .025 | < 0.0001 | 0.797 | 0.725 |
| **20:4 n-6 (AA)** | 20.3±  .643 | 18.2±  .535 | 24.9±  .233 | 24.6±  .228 | 10.5±  .346 | 10.1±  .471 | < 0.0001 | 0.020 | 0.095 |
| **20:5** | .540±  .048 | .898±  .020 | 0±0 | 0±0 | .387±  .077 | .755±  .028 | < 0.0001 | < 0.0001 | 0.0002 |
| **22:5 n-3** | 1.69±  .502 | 3.34±  .105 | 1.65±  .114 | 2.23±  .091 | .771±  .044 | 1.14±  .027 | < 0.0001 | 0.0001 | 0.020 |
| **22:6 n-3 (DHA)** | 14.4±  .742 | 13.2±  .560 | 10.9±  .771 | 10.7±  .528 | 31.9±  .692 | 31.4±  .802 | < 0.0001 | 0.298 | 0.756 |
| **23:0** | .261±  .005 | .259±  .024 | 1.32±  .074 | 1.52±  .050 | 0±0 | 0±0 | < 0.0001 | 0.04 | 0.02 |
| **23:1** | .201±  .028 | .127±  .006 | 4.43±  .691 | 4.22±  .335 | 0±0 | 0±0 | < 0.0001 | 0.72 | 0.95 |

Data displayed as mean area% ± SE relevant to each treatment group (diet/strain); n = 4. The p-values derived from 2-way ANOVA (representing diet, strain and interaction effects) are provided. CON, control; WES, Western; WES+DHA, Western + DHA. n-3, omega-3 polyunsaturated fatty acid; n-6, omega-6 polyunsaturated fatty acid; LA, linoleic acid; ALA, α-linolenic acid; AA, arachidonic acid; DHA, docosahexaenoic acid.
